# Supplementary material for: Imaging of electrically controlled van der Waals layer stacking in 1T-TaS2
Source: Nat Commun. 2025 Nov 21;16:10296. doi: 10.1038/s41467-025-65212-1 (PMC12639158; doi:10.1038/s41467-025-65212-1)
Supplement: Supplementary file 1 — Supplementary Information [file 41467_2025_65212_MOESM1_ESM.pdf]

Supplementary Information
Imaging of electrically controlled van der Waals layer stacking in 1T-TaS$_2$

Corinna Burri,[1,2] Nelson Hua,[1] Dario Ferreira Sanchez,[1] Wenxiang Hu,[1,2] Henry G. Bell,[1,2] Rok Venturini,[1,3] Shih-Wen Huang,[1] Aidan G. McConnell,[1,2] Faris Dizdarević,[1,2] Anže Mraz,[3,4] Damjan Svetin,[3] Benjamin Lipovšek,[5] Marko Topič,[5] Dimitrios Kazazis,[1] Gabriel Aeppli,[1,2,6] Daniel Grolimund,[1] Yasin Ekinci,[1] Dragan Mihailović,[3,4,7,*] and Simon Gerber[1,†]

[1]*PSI Center for Photon Science, Paul Scherrer Institute, 5232 Villigen PSI, Switzerland*
[2]*Laboratory for Solid State Physics and Quantum Center, ETH Zurich, 8093 Zurich, Switzerland*
[3]*Department of Complex Matter, Jozef Stefan Institute, 1000 Ljubljana, Slovenia*
[4]*CENN Nanocenter, 1000 Ljubljana, Slovenia*
[5]*Faculty for Electrical Engineering, University of Ljubljana, 1000 Ljubljana, Slovenia*
[6]*Institute of Physics, EPF Lausanne, 1015 Lausanne, Switzerland*
[7]*Faculty of Mathematics and Physics, University of Ljubljana, 1000 Ljubljana, Slovenia*
(Dated: October 23, 2025)

SUPPLEMENTARY METHODS

Fluorescence measurements

X-ray fluorescence of Ta and Au is used to locate the device and set the range of the spatially-resolved scans. The signal is captured by VIAMP-KC detectors. Even small traces of elements such as Cu from the sample holder, Ti from the binary markers on the substrate, and Ni under the electrodes are detected (Supplementary Fig. 1). Fluorescence data are fitted using the PyMCA libraries [1].

Experimental geometry constraints and reciprocal space reconstruction

In the following, we describe how we assign pixels on the detector at each X-ray energy (x_d, y_d, E) to reciprocal space coordinates (hkl).

The known parameters in this experiment are the X-ray energy, the detector orientation which was perpendicular to the incident X-ray beam and further detector specifications of the Eiger X 4M detector, such as the pixel size. In addition, we know the optimal pixel and energy of three accessible lattice peaks (004), (013), (014) and four commensurate charge-density wave (CCDW) peaks on the detector and also their theoretical (hkl) parameters [3], leading to seven known quantities. The measured peak positions are determined by fitting a Lorentzian to the projected intensities. The CCDW peaks are elongated along the out-of-plane direction, therefore only the in-plane (hk) dimensions are fitted. The unknown parameters in this setup are the relative position of the detector with respect to the sample (x, y, z) which was roughly measured and used as an initial guess, the sample orientation parameterized with angles (α, ϕ, χ) and the lattice constants of the flake (a, b, c). We define the detector orientation with Cartesian coordinates (x, y, z), pointing along the x, y pixel directions and z perpendicular to the detector surface. α denotes the angle between the surface normal of the detector and the surface normal of the flake, whereas ϕ and χ are the azimuthal and tilt sample angles, respectively. Since there are only seven measured parameters (three lattice and four CCDW reflections), we take the in-plane lattice parameters $a = b = 3.32$ Å as known [4] and only the out-of-plane lattice parameter c as unknown.

As a next step, a cost function is defined that includes all known and unknown parameters, and with which the full experimental geometry is described as

$$F(\mathbf{x}, \mathbf{y}) = \sum_{i=1}^{7} \left| \mathbf{Q}_i(\mathbf{x}, \mathbf{y}) - \mathbf{Q}_i^{\mathrm{theory}} \right|^2, \tag{1}$$

where $\mathbf{x}$ are the unknown parameters, $\mathbf{y}$ are the known parameters and the two-norm is used.

With this function, the reciprocal space coordinates of our measured peaks is compared to the values in the literature [3, 5]. The cost function is minimized with respect to $\mathbf{x}$ using a standard minimization algorithm from the SciPy optimization python library [6] which finds the experimental conditions $(\alpha, \phi, \chi, x, y, z, c) = (25.61°, -86.42°, 0.57°, -0.14\,\mathrm{m}, -0.06\,\mathrm{m}, 0.0\,\mathrm{m}, 0.6\,\mathrm{nm})$ of the unknown parameters that matches our experimental results. The resulting small errors are shown in Supplementary Table 1, meaning that there is good agreement between the measured peaks and the known structure of the material.

Once this experimental geometry is defined, the reciprocal space coordinates (hkl) are assigned to the pixels on the detector at every X-ray energy using the Laue equation.

The conversion from (x_d, y_d, E) to (hkl) leads to a curved surface in reciprocal space which is probed by the

* dragan.mihailovic@ijs.si
† simon.gerber@psi.ch

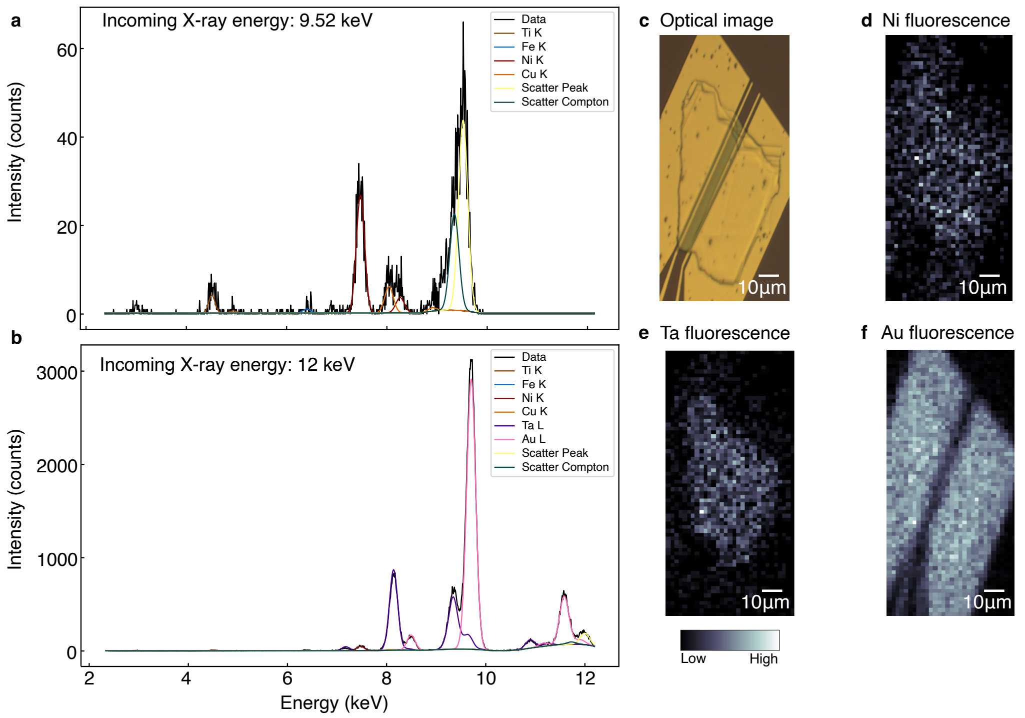

Supplementary Fig. 1. **Elements in the 1*T*-TaS$_2$ device detected by X-ray fluorescence**. **a** Total fluorescence intensity measured using an incoming X-ray energy of 9.52 keV, below the Ta *L*-edge [2]. Peak fitting traces elements such as Ti, Fe, Ni and Cu on the device and sample holder (via the respective *K*-edges), as well as elastic and Compton scattering. **b** Respective fluorescence signal measured at 12 keV, above the Ta and Au *L*-edges [2], where in addition to the elements observed in **a**, also Ta and Au are detected. **c** Optical image of the device. **d** Ni signal mapped spatially across the device showing traces along the electrodes and the flake. **e,f** The Ta and Au signals outline the flake and electrodes, respectively. The minimal intensity in d-f is 0, and the maximal intensity is 6.7 (Ni), 9.6 (Ta), and 67 counts (Au). An integration time of 100 ms was chosen for all measurements.

Peak	Literature (r.l.u)	Experiment (r.l.u)	Two-norm error (pixels)
(013) CCDW1	$(0.08, 1.23, 2.80)$	$(0.08, 1.23, 2.86)$	1.5
(013) CCDW2	$(-0.08, 0.77, 3.20)$	$(-0.08, 0.77, 3.14)$	0.7
(014) CCDW1	$(0.08, 1.23, 3.80)$	$(0.07, 1.24, 3.68)$	1.2
(014) CCDW2	$(-0.08, 0.77, 4.20)$	$(-0.08, 0.77, 4.16)$	0.7
(013) lattice	$(0, 1, 3)$	$(0.0, 1.0, 3.0)$	3.5
(004) lattice	$(0, 0, 4)$	$(0.0, 0.0, 4.0)$	5.0
(014) lattice	$(0, 1, 4)$	$(0.0, 1.0, 4.0)$	2.3

Supplementary Table 1. **Error table of the measured equilibrium peaks**. Lattice and nearby commensurate charge-density wave (CCDW) peaks detected in the experiment are listed with their index from the literature [3, 5], the calculated (hkl) positions determined from the experimental geometry and the error from the peak fits. The latter is given in pixel and is calculated by taking the norm of the absolute error divided by the reciprocal space pixel resolution of 1.03×10^{-3} reciprocal lattice units (r.l.u.).

flat 2D detector in real space. Putting all the curved surfaces of the measured energies together, allows us to reconstruct the three-dimensional (3D) shape of the lattice, CCDW, hidden state (HCDW) and dimer peaks. Supplementary Fig. 2 shows this 3D reciprocal space representation measured in the fully-switched state C. This is a volumetric plot using the intensities of the measured peaks, giving an overview over the peak shapes and positions. Importantly, it is not a spatially-resolved 3D reciprocal image, but rather the reconstruction coming from the sum of all pixels measured at the different energies. Therefore, we observe all peaks in the same plot. Next to the overview plot are extracts of reciprocal space around the lattice and CCDW peak in the unswitched A and in the fully-switched state C. We see that in the fully-switched state a HCDW peak appears below the CCDW signal, as well as an additional lattice satellite (Supplementary Discussion). For the spatially-resolved intensity

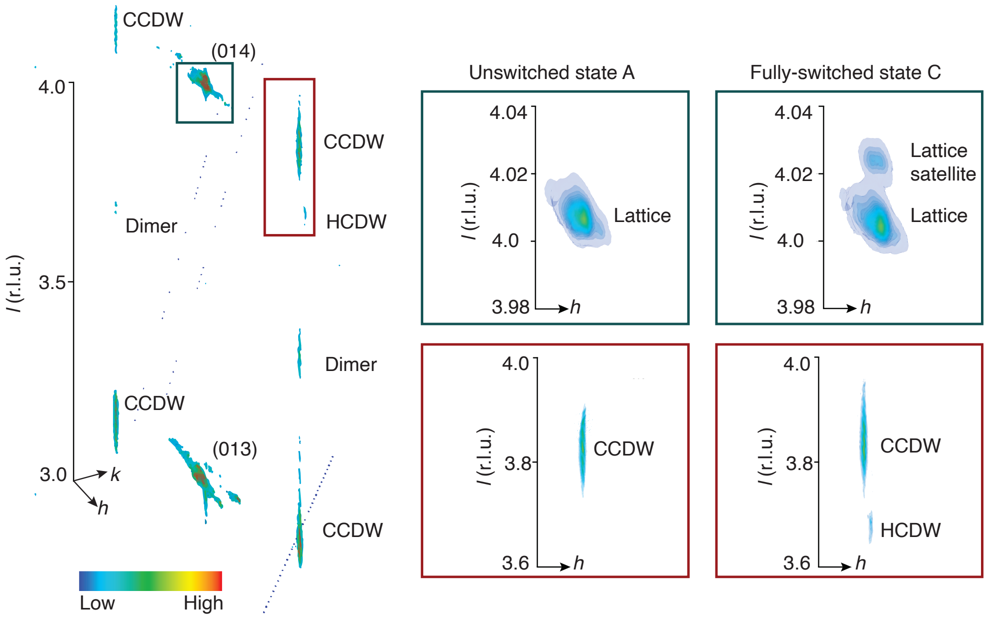

Supplementary Fig. 2. **3D reciprocal space reconstruction of the fully-switched state**. Shown on the left is the reconstruction of the fully-switched state C summed from all pixels on the flake, *i.e.* no spatial resolution, including the (013) and (014) lattice peaks, two commensurate charge-density wave (CCDW) peaks near each of them, a hidden (H)CDW peak near (014) and dimer peaks. This is a volumetric plot from the intensities of the peaks showing the general shape of the different peaks. The reconstruction is done for the measured energies between 9.2 and 12 keV, which is why only certain peaks are observed. The blue pixel lines are due to dead pixels of the detector. On the right are extracts around the (014) lattice and a corresponding CCDW peak, showing the appearance of the HCDW peak and also a new lattice satellite when comparing the unswitched A and the fully-switched state C.

maps of the device, we use such 3D reconstruction of every pixel. The peak positions are determined from the 3D reconstruction by summing along two reciprocal space directions to obtain a 1D intensity plot along the remaining reciprocal space direction and fitting a Lorentzian function to this peak.

Au ring correction

We observe five ring-like features in the raw diffraction patterns (Supplementary Fig. 3a). After the detector position with respect to the sample is determined, the 2θ angle of these rings, *i.e.* the radius r, at a certain energy of the incoming X-rays is determined using Bragg's law. Comparison with literature shows that the radii match Au with Miller indices (111), (200), (220), (311) and (222) [7] (Supplementary Fig. 3c). The electrodes are made from amorphous Au, therefore we observe these rings when spatially scanning over the device. Taking a region of interest (ROI) with radius $r \in [r_{\text{ring}} \pm 1.8]$ (in mm) corresponding to the thickness of the Au rings, allows us to collect and sum up the intensity of all detector pixels within this ROI.

This procedure is performed for every pixel (x_d and y_d) on the sample which gives us spatially-resolved intensity maps outlining the electrodes of the device. We use these electrode images for aligning the spatial scans at the different X-ray energies.

After confirming that no peak of interest cuts through the Au rings, we replace them for the 3D reciprocal space reconstruction by an average background (Supplementary Fig. 3b). This is simply done for better visibility of the peak shapes in the reconstruction, but does not affect the reconstruction itself.

Spatially-resolved maps

In the following, we describe how we obtain the spatially-resolved intensity maps and projections in reciprocal space. For each state of the device, *i.e.* at room-temperature, as well as the unswitched A, partially- B and fully-switched states C at the base temperature of 6 K, we measure spatial maps at different energies. As a next step, we perform a spatial jitter correction. That is, we spatially align the scans at different energies and for the different states. We do this by choosing a Au ring which shows clear outlines of the electrodes in the spatial maps. For each state, we first chose one reference spatial map I_{ref} measured at a certain X-ray energy. We then align all other spatial maps measured at different energies to I_{ref}. Due to the finite scan range, we confine a possible shift in the x and y spatial directions

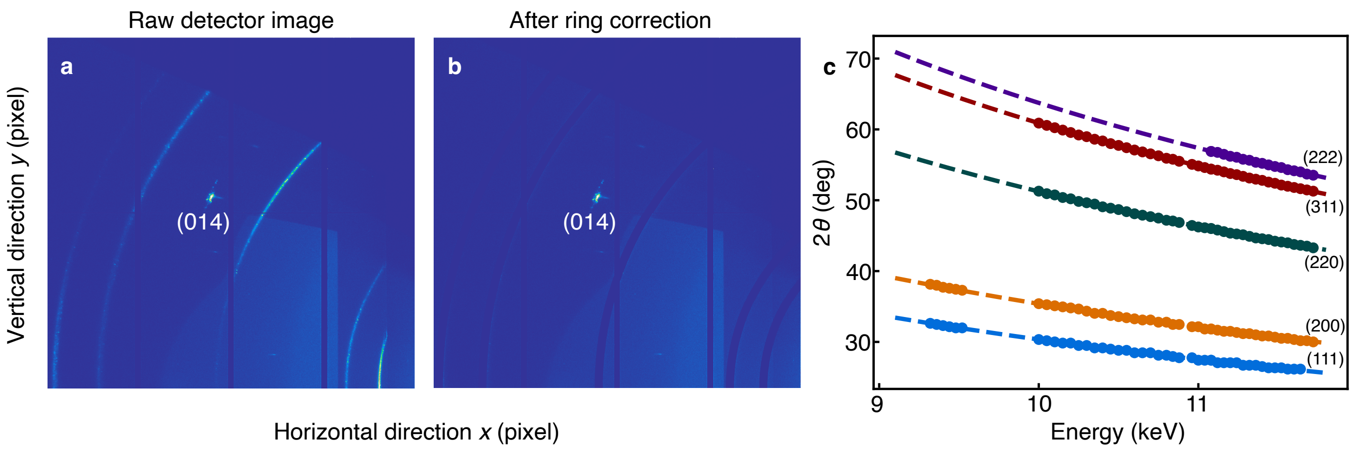

Supplementary Fig. 3. **Au ring correction**. **a** Exemplary raw detector image taken at 11.12 keV showing five rings coming from the Au electrode material, as well as the (014) lattice peak of the $1T$-TaS_2 flake. **b** The same detector image after fitting the Au rings and replacing them with an average background. **c** Photon-energy dependent scattering angle 2θ of the five Au rings observed in the experiment (data points), overlaid with the calculated corresponding (111), (200), (220), (311) and (222) Au reflections [7] (dashed lines).

to $\delta x \in [\pm 10]$ and $\delta y \in [\pm 20]$ (in pixel) and find $(\delta x, \delta y)$ such that

$$F(\delta x, \delta y) = \sum_{i,j} \left[I_{\mathrm{ref}}\left(x_i, y_j\right) - I\left(x_i + \delta x, y_j + \delta y\right)\right]^2, \quad (2)$$

summing over the pixel indices i, j, is minimized. The result shows in general good alignment within ± 1 pixel. But there are a few scans at certain energies which still have to be manually tweaked to improve the alignment.

After having the scans at different energies for every state spatially aligned and a 3D reciprocal space reconstruction for each pixel performed, we define 3D ROIs in reciprocal space around the peaks of interest. Since the CCDW and dimer peaks are elongated along l, as has been reported before [5], we take cylindrical ROIs around these peaks. For the lattice and HCDW peaks which are better defined along the out-of-plane direction, we take spherical ROIs. We compute the signal within these ROIs by summing up the intensities. For every spatial map at every energy, we set a $1.5 \times 1.5\,\mu\mathrm{m}^2$ (room temperature) and $1 \times 1\,\mu\mathrm{m}^2$ (6 K) grid according to the minimal step size of the scan. Then, we assign intensity values to the grid by considering that if the closest scan to a respective grid pixel is > 2 pixel, we set it to zero and otherwise we consider the data within the predefined ROI in reciprocal space. For the spatially-resolved intensity maps, such as in Fig. 2 of the main text, the intensity in the spatial pixels is therefore the mean of the intensities from measurements that contribute to the ROI. We note that with a minimal step size in the x and y direction of 1.5 µm at room temperature and 1 µm at the base temperature, we are in fact oversampling given the X-ray spot size of $1.5 \times 2.5\,\mu\mathrm{m}^2$ (vertical $\times$ horizontal).

After identifying the region which switched to the HCDW state from the spatial maps, we can subdivide the device into different spatial regions to extract the spatially-resolved 2D reciprocal space projections (Fig. 3 of the main text) and calculate the ratio of the HCDW and CCDW peak intensity. Then, we sum up the 3D reciprocal space reconstructions of pixels within these regions. For the projected maps, such as Fig. 3a-d of the main text, we set a grid in reciprocal space and interpolate. From this, we obtain the 2D projections by summing along one reciprocal space axis and dividing by the number of pixels in the spatial region.

SUPPLEMENTARY DISCUSSION

Room-temperature spatial maps

At the beginning of the experiment, the device is at room temperature and spatial scans at different X-ray energies are measured to check the alignment, as well as to record the lattice and the nearly-commensurate (NC)CDW signals. We take spatially-resolved scans at a few energies, corresponding to selected cuts through reciprocal space. Hence, we only observe a few peaks with reduced statistics compared to the low-temperature measurements, resulting in a less accurate 3D reconstruction. Nevertheless, we take 3D ROIs around the (013) lattice peak and a respective NCCDW peak to plot the corresponding spatial intensity map which both reproduce the general shape of the flake (Supplementary Fig. 4).

Dimers and phase switching

As discussed in [5, 8], dimer peaks associated with a doubling of the real-space unit cell in the out-of-plane

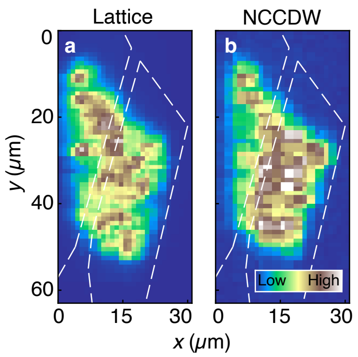

Supplementary Fig. 4. **Room-temperature spatially-resolved maps**. **a** and **b** show the (013) lattice and nearly commensurate charge-density wave (NCCDW) peak intensities, respectively. The minimal mean intensity is 0 and the maxima are 2.0 (013) and 0.2 (NCCDW) counts for an integration time of 100 ms. Dashed lines outline the electrodes. The step size is 1.5 µm.

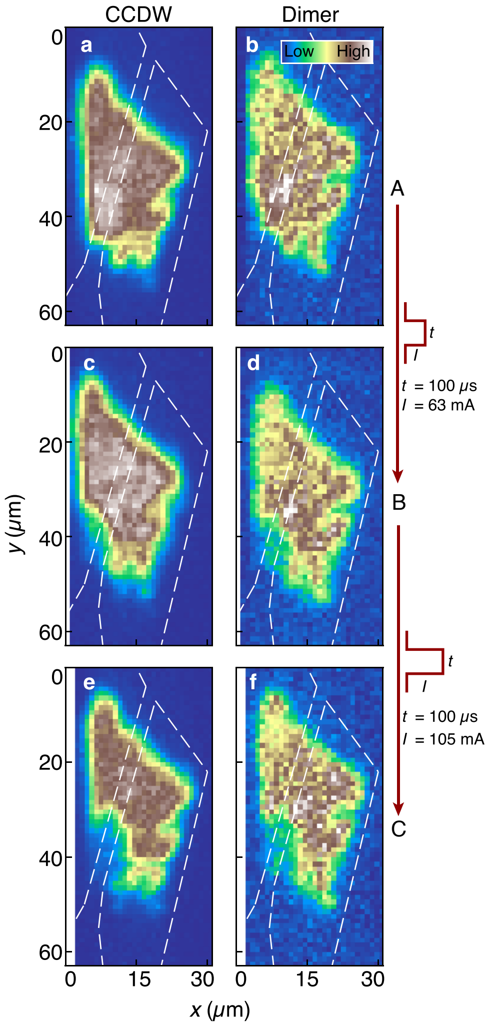

Supplementary Fig. 5. **Vanishing dimers in the switching region**. **a** and **b** show the spatially-resolved intensities of the commensurate charge-density wave (CCDW) and dimer peaks, respectively, at the base temperature of 6 K in the unswitched state A. **c**,**d** and **e**,**f** show the maps in the partially- B and fully-switched state C, respectively, where both signals vanish in the switching region. The minimal mean intensity is 0 and the maxima are $5 \cdot 10^{-2}$ (CCDW) and $5 \cdot 10^{-3}$ (dimer) counts for an integration time of 100 ms. Dashed lines outline the electrodes. The step size is 1 µm.

direction were observed in the CCDW state and vanish upon switching to the optically-induced (o-)HCDW state. We also observe dimer peaks in our electrically-switched measurements (Supplementary Fig. 2) and take 3D ROIs around those in reciprocal space. We record only a few energy scans cutting through the dimer peaks, meaning that the statistics is reduced compared to other peaks. Supplementary Fig. 5 shows the spatially-resolved intensity maps of the CCDW and dimer peaks in the unswitched A, the partially- B and the fully-switched state C. We see that both peak intensities vanish in the bottom left part of the flake in the fully-switched state. Hence, we observe the same vanishing of dimer peaks in the electrically-induced (e-)HCDW state in the inter-gap region as reported for optical switching, meaning for both types of excitation there is also collapse of dimerization. Indirectly, observed as a local doubling of the out-of-plane dispersion, this has also been reported in a recent μARPES study [9]. As addressed in the main text, we observe that the intensities of the CCDW, as well as the dimer peaks in the unswitched state, are the highest in the bottom left part of the flake that also switches to the HCDW state. This supports the notion that the location of the switching region may not only be dictated by the geometry of the device, but also the most homogeneous (dimerized) CDW order.

Switching-induced out-of-plane lattice contraction

As reported before for the o-HCDW state [10], we also observe a change of the out-of-plane lattice constant in the e-HCDW state. Supplementary Fig. 6 shows the 1D projection onto the out-of-plane reciprocal space coordinate l of the (004) lattice peak. The signal is measured on the entire flake (no spatial resolution) in the unswitched A, the partially- B and the fully-switched state C. The high intensity peak is the (004) lattice peak reflection. We note that because this measurement is not spatially-resolved, intensity (from the unswitched portions of the flake) still shows up there even in the C state. In the B and C states a satellite lattice peak emerges. Thus, we observe that for the HCDW state the out-of-plane lattice peak position shifts to a higher reciprocal space value, meaning that the lattice contracts by ≈0.5 %. The out-of-plane lattice constant difference between the switched and unswitched region leads to strain along the border which we speculate facilitates the switching in-plane but also in volume. Similarly, also the

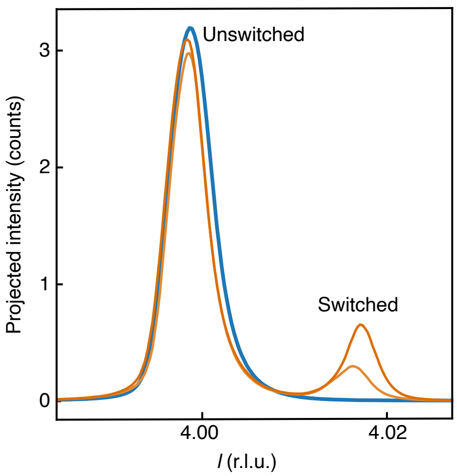

Supplementary Fig. 6. **Out-of-plane lattice contraction**. Projections of the (004) lattice peak along the reciprocal space l direction in the unswitched (A, blue), the partially- (B, light orange) and the fully-switched state (C, dark orange). This measurement integrates spatially over the entire flake and uses an acquisition time of 100 ms. Upon switching intensity shifts from the (004) peak to the satellite at higher l values, corresponding to an out-of-plane lattice contraction of ≈0.5 %.

c lattice constant contracts between the NCCDW and CCDW states [8, 11–13].

We set two ROIs around the lattice signals in reciprocal space: $l \in [3.99, 4.01]$ and $[4.015, 4.035]$ r.l.u. for the main and satellite peaks, respectively, to obtain spatial maps by summing up the intensities within these ROIs (Supplementary Fig. 7). Also here we observe that the HCDW state switching region appears at the bottom left corner of the flake. Thus, not only the electronic CDW structure is modified in the hidden state but also the out-of-plane lattice contracts in the non-volatile switching region of the device. In the following, we show the agreement of the switching observed from the out-of-plane lattice contraction and the HCDW peak.

As done for Fig. 3 of the main text, we define different spatial regions of the device: outside of the switching region (light blue), near the switching region (light purple) and in-between the electrodes (dark purple). We then map out the 2D reciprocal space projections of the (004) lattice peak (Supplementary Fig. 8). Unlike the CCDW peak which shows a characteristic elongation, the lattice peak is sharp also in the out-of-plane direction. The correlation of the ratio of the projected out-of-plane lattice intensities of the main and satellite signal, $I_{\text{satellite}}/I_{\text{main}}$, and that of the HCDW and CCDW peak intensity, $I_{\text{HCDW}}/I_{\text{CCDW}}$, is shown in Supplementary Fig. 9a. Supplementary Fig. 9b compares the contours of the switching region based on the ratio of the lattice signals, and the HCDW/CCDW peaks, revealing that the two are also spatially correlated. Thus, also the lattice peak contraction can be used as a fingerprint to locate the HCDW switching.

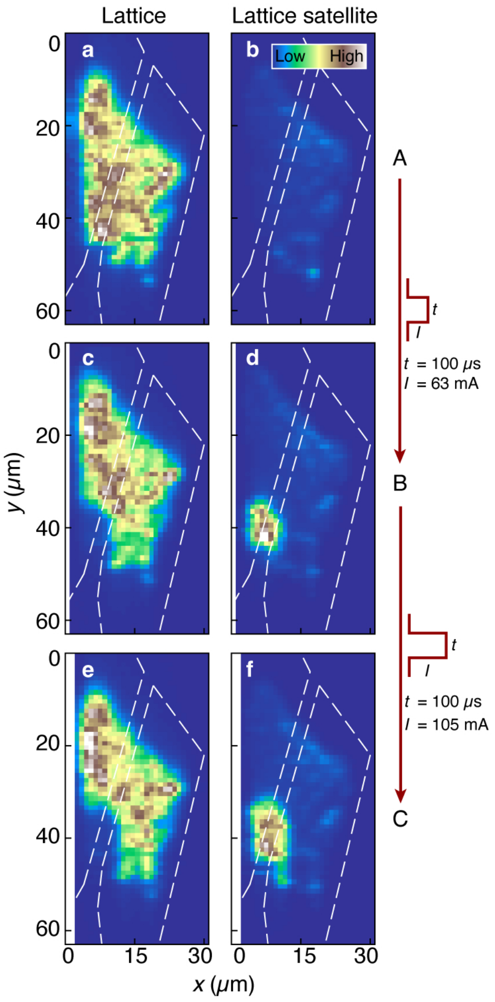

Supplementary Fig. 7. **Spatially-resolved out-of-plane lattice contraction**. **a** and **b** show intensity maps at the (004) lattice and satellite peak positions, respectively, in the unswitched state A. **c**,**d** and **e**,**f** show the corresponding maps in the partially- B and fully-switched state C, respectively. As for the CCDW and HCDW signal, the main lattice peak vanishes in the bottom left part of the flake where the lattice satellite peak is observed instead, showing that the switching region can also be observed via the out-of-plane lattice contraction. Color scales show a minimal mean intensity of 0 and a maximum of 5 counts for an integration time of 100 ms. Dashed lines outline the electrodes. The step size is 1 μm.

Finite element method simulations

Numerical simulations are performed using the COMSOL Multiphysics software, which uses the finite element method to solve partial differential equations governing heat conduction in solid materials and devices:

$$\rho C_{\mathrm{p}} \frac{\partial T}{\partial t} - \nabla \cdot (k \nabla T) = Q_{\mathrm{e}}, \tag{3}$$

$$Q_{\mathrm{e}} = \sigma |\nabla V|^2, \tag{4}$$

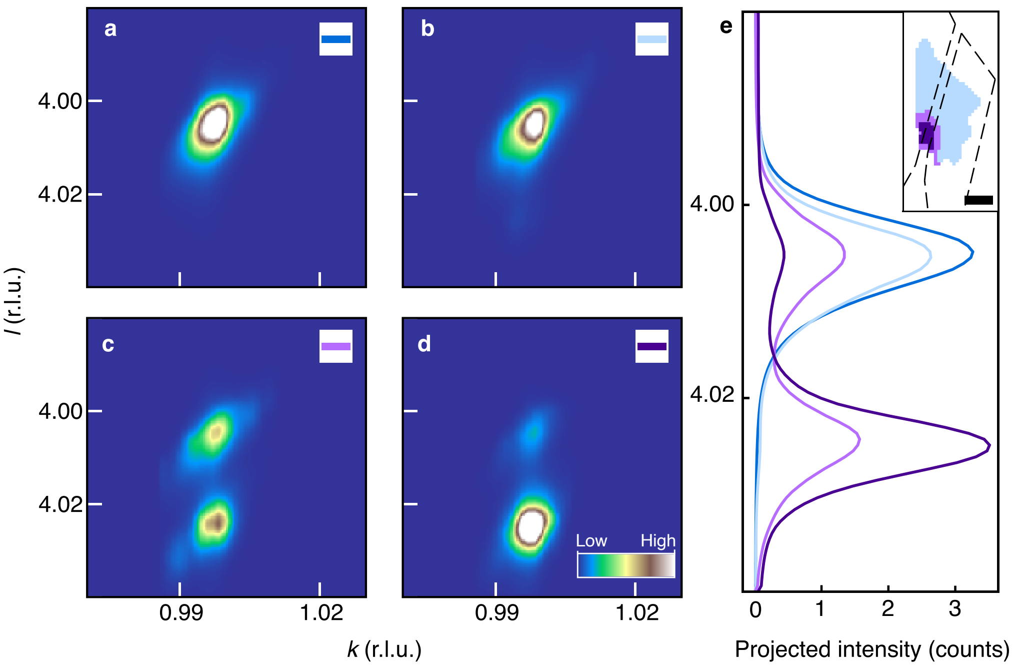

Supplementary Fig. 8. **Momentum- and real-space structure of the lattice contraction**. **a** 2D (kl) reciprocal space projection of the lattice peak in the unswitched state (A, dark blue). **b-d** Corresponding measurement in the fully-switched state C in the light blue, as well as the light and dark purple region (inset of e), respectively. The 2D projected intensities are normalized by the size of the respective spatial region. **e** Out-of-plane projection of the averaged intensities shown in a-d for an integration time of 100 ms. The inset shows the color-coded regions on the flake. Dashed lines in the inset outline the electrodes. The scale bar is 20 µm.

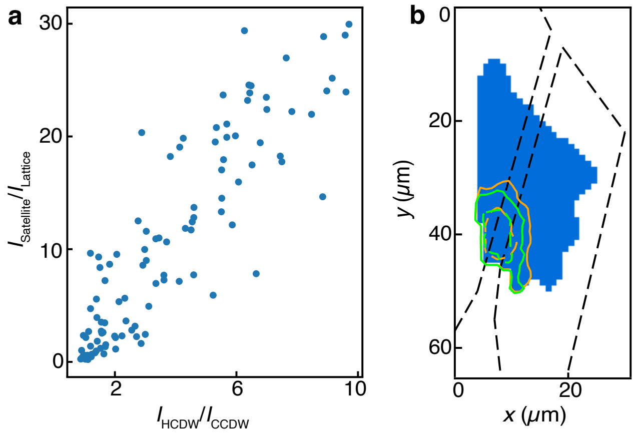

Supplementary Fig. 9. **Correlation between lattice contraction and HCDW peak appearance**. **a** Linear correlation among the ratio of the out-of-plane lattice signals and the hidden/commensurate charge-density wave (H/C)CDW intensities. **b** The blue region indicates the shape of the flake. Contours of the spatial switching region identified by the lattice contraction and HCDW appearance are shown in orange and green, respectively. Dashed lines outline the electrodes.

where T is the temperature, V the volume and Q_e the heat source term. The density of the material ρ and specific heat capacity C_p [14], as well as the k thermal [15] and σ electrical conductivity [16, 17] of all materials in the device are taken from the literature or our own experiments. The thermal model is coupled to the electromagnetic one which provides Q_e in the differential equations by calculating the resistive losses in the device, *i.e.* Joule heating.

The structure and dimensions of the simulated device is shown in Supplementary Fig. 10a. The geometry of the Au contacts and the $1T$-TaS_2 crystal flake is replicated both in shape and size, including irregularities, such as the narrower spacing at one (the left) side of the structure and the two additional disconnected contact fingers in the middle. Excitation of the device is applied via the two outer contacts by assuming 100 µs square wave current pulses of amplitudes up to 10 mA. At the beginning of each simulation, the device is fully in the unswitched state A and a temperature of 4 K is assumed. After applying the excitation, switching of the elements from A to the fully-switched state C is handled by a simplified model which predicts formation of the HCDW state domain wall network under the influence of a sufficiently large charge carrier injection. Thus, in the simulations the state of each element is switched once the current density of the element reaches a predefined current density threshold of 5×10^8 A/m^2, calibrated by the experiment. Supplementary Fig. 10b shows the switched material (orange) for different magnitudes of current excitation. All cases identify the preferential area of device

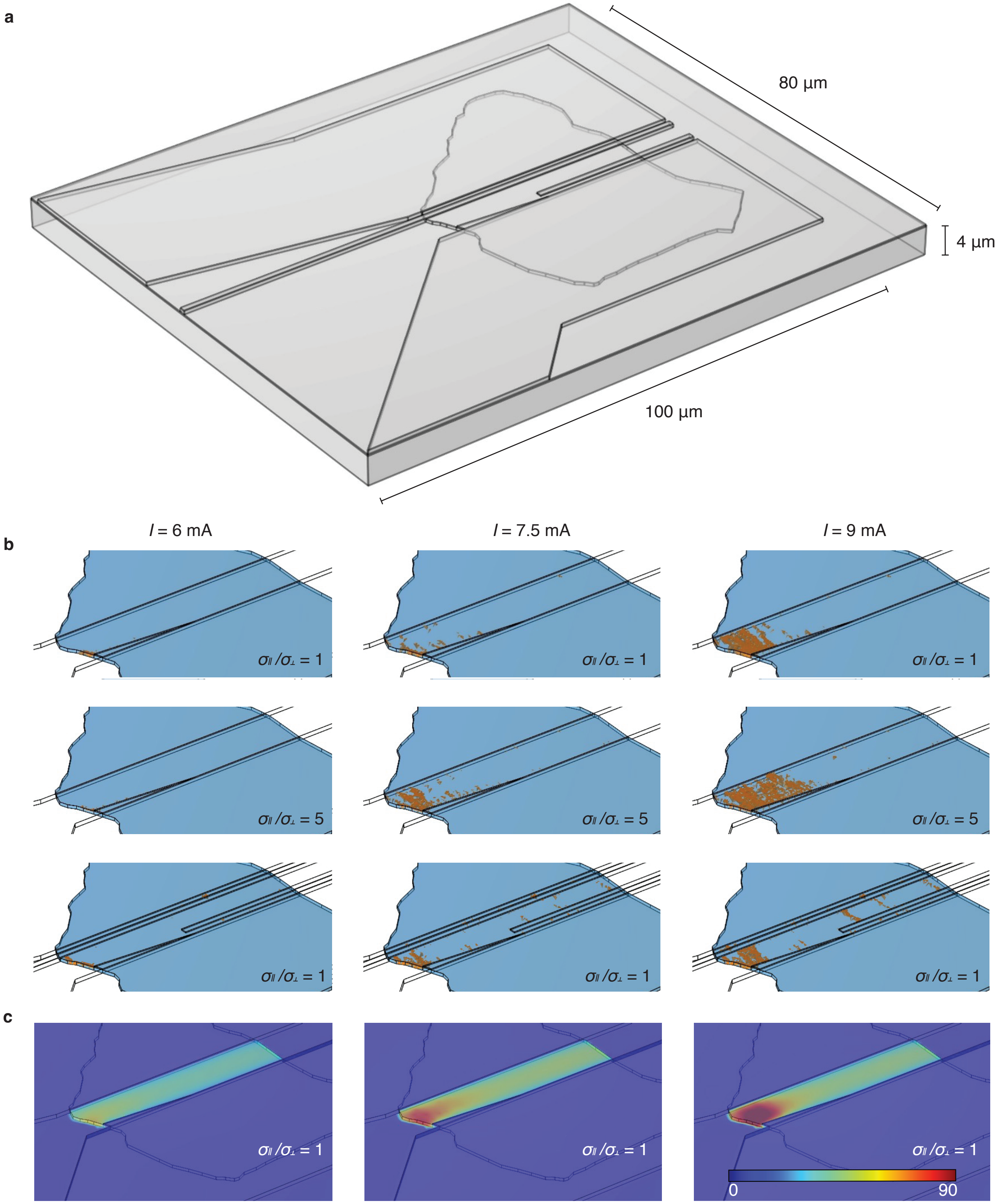

Supplementary Fig. 10. **Finite element method simulations**. **a** Schematic of the device considered for the simulation. **b** Switching volume (orange) under different current excitation levels (columns), as well as assuming different material anisotropy $\sigma_{\|}/\sigma_{\perp}$ (top and middle row) and inclusion of a more detailed electrode geometry (bottom row). **c** Device heating upon switching for the simulations shown in the top row of b (color scale in Kelvin).

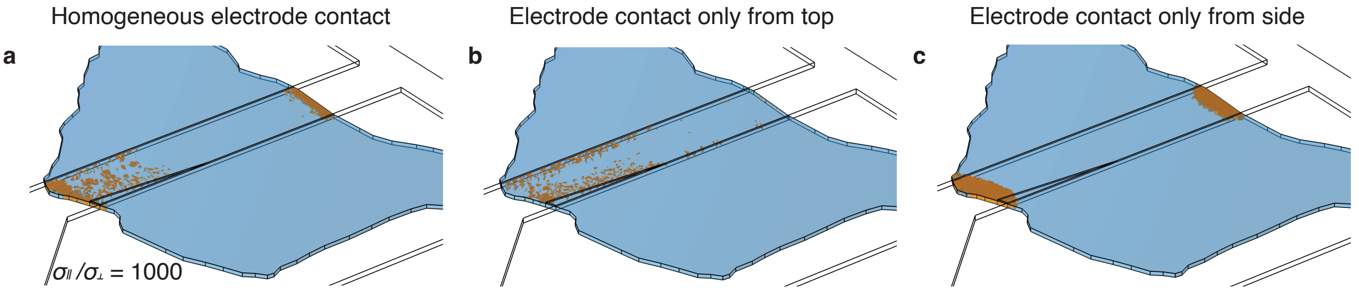

Supplementary Fig. 11. **Influence of the conductivity anisotropy and electrode contact location.** The switching volume (orange) for a current excitation of $I = 9$ mA and conductivity anisotropy of $\sigma_{\parallel}/\sigma_{\perp} = 1000$ is shown. **a** considers homogeneous electrode contact, whereas **b** and **c** assume contact only from the top and side, respectively.

switching at the left side of the device, which is influenced primarily by the narrower spacing between the contacts. With increasing current amplitude, the area gradually expands along the intergap space. Furthermore, we observe that the penetration depth of the switching is determined by the assumed level of anisotropy of $1T$-TaS_2. In the isotropic case (Supplementary Fig. 10b, top row), the switched volume propagates throughout the entire crystal flake, whereas it remains closer to the surface when assuming an out/in-plane conductivity anisotropy of 1:5 (Supplementary Fig. 10b, middle row). A further set of simulations (Supplementary Fig. 10b, bottom row) tests also the influence of the disconnected contact fingers. While they do affect the quantitative extent of the switched volume, the additional conductive paths they provide qualitative agree with the simulations in the top row of Supplementary Fig. 10b. However, contrary to the experiment the simulations do not reproduce the lateral and vertical expansion of the switched region beneath the contacts.

To test the influence of the conductivity anisotropy, we also perform simulations using a higher anisotropy ratio of 1:1000 (Supplementary Fig. 11), as reported in Ref. [18]. Assuming homogeneous electrode contact, switching appears on both sides of the flake. When separating the contact contributions—considering only top or side contact—we observe that side contacts can induce switching beneath the electrodes, but also trigger switching on both sides of the flake. In reality, however, the electrode contact is expected to be largely homogeneous due to the 160-nm-thick Au layer. But even with a high conductivity anisotropy and variations of the electrode contact, we cannot reproduce the experimentally observed localized switching confined to one side of the flake and extending well beneath the electrodes. This suggests that while the conductivity anisotropy and contact geometry may play a role in determining the switching location, they are insufficient to explain the behavior observed in the experiment and, consequently, additional mechanisms must be at play.

We speculate that propagation of local strain between the switched and unswitched area—a feature presently not included in the numerical simulations but measured in the experiment (Supplementary Fig. 7)—is responsible for the lateral extent of the switching area under the electrodes. We also observe that compared to the experiment, the device switches fully at a lower current amplitude (between 10 to 20 mA), which we attribute to slight discrepancies between the assumed and effective material parameters. Finally, in Supplementary Fig. 10c device heating due to the current excitation is also demonstrated for the isotropic situation (Supplementary Fig. 10b, top row). The heating upon current pulse application localized to the left side of the device is also in agreement with the preferential path of the current flow across the $1T$-TaS_2 flake.

SUPPLEMENTARY REFERENCES

[1] V. Solé *et al.*, A multiplatform code for the analysis of energy-dispersive X-ray fluorescence spectra, Spectrochim. Acta Part B At. Spectrosc. **62**, 63 (2007).

[2] A. Thompson, *X-ray Data Booklet* (LBNL, UC, 2001).

[3] C. Laulhé *et al.*, X-ray study of femtosecond structural dynamics in the 2D charge density wave compound $1T$-TaS_2, Physica B: Cond. Matter **460**, 100 (2015).

[4] F. Jellinek, The system tantalum-sulfur, J. Less-Common Met. **4**, 9 (1962).

[5] Q. Stahl *et al.*, Collapse of layer dimerization in the photo-induced hidden state of $1T$-TaS_2, Nat. Commun. **11**, 1247 (2020).

[6] P. Virtanen *et al.*, SciPy 1.0: fundamental algorithms for scientific computing in Python, Nat. Methods **17**, 261 (2020).

[7] M. Graf *et al.*, X-ray studies of nanoporous gold: Powder diffraction by large crystals with small holes, Phys. Rev. Mater. **1**, 076003 (2017).

[8] Y. D. Wang *et al.*, Band insulator to Mott insulator transition in $1T$-TaS_2, Nat. Commun. **11**, 4215 (2020).

[9] Y. Nitzav *et al.*, Emergence of a Fermi-surface in the current-driven hidden state of $1T$-TaS_2, `https://arxiv.org/abs/2407.05535`, (2024).

[10] I. Vaskivskyi *et al.*, A high-efficiency programmable modulator for extreme ultraviolet light with nanometre feature size based on an electronic phase transition, Nat. Photon. **18**, 458 (2024).

[11] F. I. Givens and G. E. Fredericks, Thermal expansion

of $NbSe_2$ and TaS_2, Phys. Chem. Solids **38**, 13634365 (1977).

[12] O. Sezerman *et al.*, Thermal expansion of $1T$-TaS_2 and 2H-$NbSe_2$, Solid State Commun. **36**, 737 (1980).

[13] D. R. P. Guy *et al.*, High pressure investigation of the CDW phase diagram of $1T$-TaS_2, in *Charge Density Waves in Solids*, Vol. 217 (1985) pp. 80–83.

[14] A. Suzuki *et al.*, Thermal evidences for successive CDW phase transitions in $1T$-TaS_2, Solid State Commun. **53**, 201 (1985).

[15] M. D. Nunez-Regueiro *et al.*, Thermal conductivity of $1T$-TaS_2 and $1H$-$TaSe_2$, Phys. Rev. Lett. **55**, 1931 (1985).

[16] L. Stojchevska *et al.*, Ultrafast switching to a stable hidden quantum state in an electronic crystal, Science **344**, 177 (2014).

[17] D. Mihailovic *et al.*, Ultrafast non-thermal and thermal switching in charge configuration memory devices based on $1T$-TaS_2, Appl. Phys. Lett. **119**, 013106 (2021).

[18] D. Svetin *et al.*, Three-dimensional resistivity and switching between correlated electronic states in $1T$-TaS_2, Sci. Rep. **7**, 46048 (2017).
